# Based on Proteomics Data Revealing the Potential of Traditional Chinese Medicine in Treating Irritable Bowel Syndrome

**DOI:** 10.1155/mi/7748351

**Published:** 2025-07-23

**Authors:** Yizhan Wu, Fei Guo, Xiaoxia Xu, Ya Liu, Jiangwei Liu

**Affiliations:** ^1^Department of Graduate School, Xinjiang Medical University, Urumqi 830000, Xinjiang Uygur Autonomous Region, China; ^2^Department of Emergency Trauma Surgery, The First Affiliated Hospital of Xinjiang Medical University, Urumqi 830054, Xinjiang Uygur Autonomous Region, China; ^3^Department of Proctology, General Hospital of Xinjiang Military Command, Urumqi 830000, China; ^4^Department of Rehabilitation, Daping Hospital, Army Medical University, Chongqing 400042, China; ^5^Key Laboratory of Special Environmental Medicine of Xinjiang, General Hospital of Xinjiang Military Command, Urumqi 830000, Xinjiang Uygur Autonomous Region, China

**Keywords:** irritable bowel syndrome, molecular dynamics simulation, network pharmacology, proteomics, traditional Chinese medicine

## Abstract

**Introduction:** Irritable bowel syndrome (IBS) is a chronic functional gastrointestinal disorder characterized by abdominal pain and altered bowel habits. Despite its high prevalence, the etiology of IBS remains elusive, and there is an unmet need for targeted therapeutic interventions.

**Material and Methods:** We initiated our study by conducting a causal analysis of proteomics data from 2941 proteins associated with IBS. Following this, we performed enrichment analysis to identify pathways and processes that may be implicated in the etiology of IBS. Subsequently, we utilized network pharmacology to explore the active compounds in traditional Chinese medicine that target the core proteins identified in our analysis. Molecular docking and molecular dynamics (MD) simulation are used to assess the stability of compound-protein binding.

**Results:** Our research has identified 169 proteins that have a positive causal relationship with IBS. We found that pathways linked to viruses, immune cells, and cytokines might play a role in IBS. Two traditional Chinese medicines, Phellodendri Amurensis Cortex (PAC) and Achyranthis Bidentatae Radix (ABR), showed potential in treating IBS, possibly through active compounds like quercetin, berberine, and evodiamine, targeting proteins Tumor Protein p53 (TP53), 5′(3′)-Deoxyribonuclease (NT5E), Jun Proto-Oncogene (JUN), and major histocompatibility complex (MHC), Class II Invariant Chain (CD74). Additionally, we conducted molecular docking and MD simulations, and the results indicated that each protein has good binding stability with its corresponding compound.

**Conclusion:** These findings not only deepen our understanding of the pathophysiological mechanisms of IBS but also indicate potential molecular targets for the development of innovative treatment strategies while highlighting the broad application prospects of traditional Chinese medicine in the field of IBS treatment.

## 1. Introduction

Irritable bowel syndrome (IBS) is a functional bowel disorder characterized by recurrent abdominal pain and changes in bowel habits, such as diarrhea, constipation, or alternating between the two, without any organic lesions found upon intestinal examination [1]. Additionally, many patients experience nonpainful abdominal discomfort, psychological symptoms (such as anxiety and depression), and other symptoms related to visceral and somatic pain [2]. Currently, the exact etiology of IBS is not fully understood, but research indicates that imbalances in the gut microbiota, abnormal gut–brain interactions, increased visceral sensitivity, changes in colonic motility, and psychological factors may play a key role in the development of IBS [3, 4]. Although the exact pathophysiological mechanisms of IBS are not fully understood, leading to treatments that mainly focus on symptom relief with limited effectiveness, there is a growing global interest in the clinical effects of traditional Chinese medicine [5]. Network meta-analyses have shown that both traditional Chinese medicines and antispasmodic drugs have certain efficacy in improving IBS symptoms and reducing abdominal pain [6]. However, the relatively high incidence of adverse events associated with traditional Chinese medicines cannot be ignored [6]. Further meta-analyses and trial sequential analyses (TSAs) have also confirmed the effectiveness of traditional Chinese medicines in relieving IBS symptoms, but also pointed out that their adverse event rate is higher than that of placebos [7]. In addition, a network meta-analysis of randomized controlled trials emphasized that traditional Chinese medicines may be beneficial for the clinical symptom relief of IBS patients and are recommended as an alternative therapy [8]. Synthesizing these findings, while traditional Chinese medicine shows potential in the treatment of IBS, its safety issues cannot be overlooked. Therefore, it is particularly necessary to continue searching for and researching traditional Chinese medicines for IBS, in the hope of discovering safer and more effective treatment plans.

Proteomics is a science focused on proteins within organisms, covering their composition, structure, function, and interactions. By meticulously analyzing protein expression patterns in cells or tissues, proteomics can reveal changes in the proteome during different biological processes (BPs) and disease states. For the complex disease of IBS, the application of proteomics may help identify biomarkers closely related to the development of IBS and further elucidate its molecular pathological mechanisms. The application of this technology not only provides the possibility for early diagnosis and precise typing of IBS but also lays the foundation for the development of new therapeutic targets and strategies, thus promoting the advancement and innovation of IBS treatment methods.

Mendelian randomization (MR) is an innovative method for causal inference that uses genetic variations as instrumental variables (IVs) to explore the potential impact of modifiable exposure factors on health, development, or social outcomes in a manner akin to random assignment [9]. A key advantage of MR is that it utilizes the natural experimental characteristic of genetic variations being randomly assigned to offspring at conception, thereby reducing common confounding biases and reverse causality in traditional observational studies, making it a rigorous approach to revealing causal relationships [10].

Network pharmacology is an interdisciplinary research field that integrates knowledge from pharmacology, systems biology, network science, and informatics, providing us with a novel perspective—exploring the mechanisms of drug action and their interactions from a holistic and systematic level. This approach comprehensively reveals the intricate network connections between drugs, targets, and diseases and predicts the potential efficacy and actions of drugs, paving new avenues for drug research and clinical applications.

**Figure 1:**
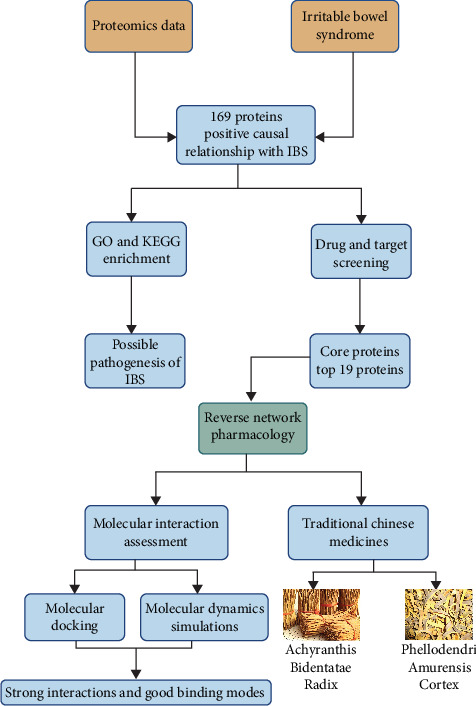
Graphical abstract. GO, Gene Ontology; IBS, irritable bowel syndrome; KEGG, Kyoto Encyclopedia of Genes and Genomes.

In this study, we utilized proteomics data and applied MR methods to delve into the pathogenesis of IBS. Subsequently, we employed network pharmacology approaches to perform a reverse screening of drugs that may target the core therapeutic targets of IBS, with the aim of discovering potential therapeutic agents for the treatment of IBS.  Figure 1 illustrates the workflow of this study.

## 2. Methods

### 2.1. MR Study Design

In this study, we adhere to a two-way MR approach to ensure the rigor of the data [11]. This process includes conducting forward MR with proteomics data as the exposure and IBS as the outcome. Additionally, we conducted an MR analysis with IBS as the exposure and proteomic data as the outcome. Through this, we eliminated proteins that have a bidirectional causal relationship with IBS, retaining only those proteins that have a positive causal relationship with IBS. This study strictly follows the basic assumptions of MR: (1) Relevance Assumption: The genetic variants used as IVs must have a reliable association with the proteins; (2) Independence Assumption: Any association between genetic variants and IBS should be mediated through the proteins and not through other pathways; (3) Exclusion Restriction Assumption: Genetic variants can only affect IBS through proteins and not through any other direct causal pathways [12].

### 2.2. Proteomics Data Source

The proteomics data utilized in this study were derived from the “DF10 v1 proteomics QTL results” dataset released by the FinnGen database in April 2024 (URL: https://www.finngen.fi/en/access_results). This dataset encompasses Protein Quantitative Trait Loci (pQTL) information for 2941 proteins, providing a rich resource of genetic IVs for MR research. After completing the download of the raw proteomics data, we first performed data conversion to obtain unique identifiers (IDs) corresponding to each single-nucleotide polymorphism (SNP).

### 2.3. IBS Data Source

In this study, we utilized data on IBS, sourced from the GWAS Catalog database (URL: https://www.ebi.ac.uk/gwas/home), under the specific ID: GCST90129446. The dataset encompasses 28,419 European ancestry cases and 182,876 European ancestry controls. ZorinaLichtenwalter et al. [13] in the journal PAIN in May 2023 originally published these data. As our research solely relies on publicly accessible database information, and given that ethical approval for all participants involved in the original study was already secured, no additional ethical review was required for the present investigation.

### 2.4. Filters for IVs

Linkage disequilibrium refers to the association between alleles at two or more loci within a population, which can influence the estimation of the relationship between genetic variations and exposure and outcome. Eliminating linkage disequilibrium helps to improve the accuracy of causal inference, ensuring that the selected genetic variations (as IVs) are more likely to affect the outcome only through their association with the exposure [9].

In this study, we screened the pQTL data to ensure that the selected SNPs had a strong enough association with the proteins. First, we conducted a preliminary screening based on a criterion of *p*-value less than 1e–5. Subsequently, we set a linkage disequilibrium threshold of *r*^2^ < 0.1 within a 100-kilobase clumping window [14]. For IBS-related SNPs, we adopted more stringent selection criteria, retaining only SNPs with *p*-values less than 5e–6 [15]. To maintain the independence of IVs, we set a linkage disequilibrium threshold of *r*^2^ less than 0.001 within a 10,000 clumping window [16].

In the MR analysis, we only considered SNPs with an *F*-statistic greater than 10 as strong IVs [17]. This rigorous selection process ensures that our research results are highly reliable and valid.

### 2.5. Statistical Analysis

In conducting the forward MR analysis, we initially utilized the inverse variance weighted (IVW) method to select significant results, with a criterion of *p*-value less than 0.05 [18]. Subsequently, we performed a reverse analysis with IBS as the exposure factor, again selecting significant results with a *p*-value less than 0.05 and excluding proteins that have a reverse causal relationship with IBS [19]. To ensure the reliability of the results, we used the MR-Egger regression method to detect potential horizontal pleiotropy [20]. Furthermore, we assessed heterogeneity through Cochran's *Q* test and utilized the MR-PRESSO method to identify outliers, ensuring the accuracy of the analytical results [20]. This series of rigorous steps aids us in accurately evaluating the causal relationship between proteins and IBS.

### 2.6. Enrichment Analysis

To explore the potential pathogenic mechanisms of IBS, this study comprehensively employs Gene Ontology (GO) and Kyoto Encyclopedia of Genes and Genomes (KEGG) enrichment analyses [21, 22]. We rely on the bioinformatics online analysis platform (URL: https://www.bioinformatics.com.cn/) to conduct GO and KEGG enrichment analysis as well as the plotting of bubble plots [23, 24]. GO enrichment analysis assists in identifying BPs and molecular functions (MFs) associated with IBS, while KEGG enrichment analysis enables us to investigate the specific metabolic pathways and signaling networks involved by these genes. Through these analyses, we can gain a more comprehensive understanding of the potential molecular mechanisms of IBS and provide potential targets for future therapeutic strategies.

### 2.7. Constructing Protein–Protein Interaction (PPI) Networks and Filtering Core Proteins

We conducted a systematic analysis of the interactions between proteins identified by MR analysis using the String database (URL: https://cn.string-db.org/) [25, 26]. Subsequently, we performed a topological analysis of these protein networks using Cytoscape software (version 3.10.1). By calculating the degree values of each protein in the network, we selected core proteins closely related to IBS with a degree threshold of ≥10 [27].

### 2.8. Acquisition of Core Protein-Related Drugs

In this study, we conducted an exhaustive search for drugs related to core proteins using the TCMSP (Traditional Chinese Medicine Systems Pharmacology) database (URL: https://tcmsp-e.com/tcmsp.php) and the Herb database (URL: http://herb.ac.cn/) [28, 29]. During the screening process, we applied strict criteria, retaining only those compounds with an oral bioavailability (OB) ≥30%, a drug-likeness (DL) index ≥0.18, and a half-life (HL) ≥4 h [30]. Furthermore, we conducted an in-depth search and analysis of the traditional Chinese medicine components associated with these compounds, aiming to identify potential active ingredients and drug candidates.

### 2.9. Molecular Docking

The structures of these compounds are derived from PubChem (URL: https://pubchem.ncbi.nlm.nih.gov/), while the protein structures originate from the Protein Data Bank (URL: https://www.rcsb.org/) [31, 32]. Before docking, all compounds underwent hydrogenation and charge assignment, and the protein structures were cleaned in PyMOL 2.5.5 to remove water and nonligand molecules. Molecular docking was performed using AutoDock Vina 1.1.2 to analyze the binding of the compounds to the target proteins.

### 2.10. Molecular Dynamics (MD) Simulation

A 100 ns MD simulation of the complex was performed using Gromacs 2023. The protein was parameterized with the CHARMM 36 force field [33], and the ligand topology was constructed with the GAFF2 force field parameters. Periodic boundary conditions were applied, and the protein–ligand complex was placed in a cubic box. The TIP3P water model was used to solvate the box with water molecules [34]. Electrostatic interactions were handled using the particle mesh Ewald (PME) method and Verlet algorithm. Subsequently, the system was equilibrated in the isothermal–isobaric ensemble for 100,000 steps, with a coupling constant of 0.1 ps, over a period of 100 ps of simulation. Van der Waals and Coulombic interactions were calculated with a cutoff of 1.0 nm. Finally, the system was subjected to MD simulation under constant temperature (300 K) and pressure (1 bar) using Gromacs 2023, with a total duration of 100 ns.

## 3. Results

### 3.1. Results for MR

Utilizing MR analysis, we have successfully screened 169 proteins that are potentially closely associated with the pathogenesis of IBS, with an IVW *p*-value threshold of less than 0.05. These results have all passed tests for pleiotropy and heterogeneity, and have also passed the MR-PRESSO test. Furthermore, in the scatter plots corresponding to each protein, the trends of the results from five different MR analyses (IVW, MR Egger, weighted median, weighted mode, and simple mode) all show consistency.

### 3.2. GO- and KEGG-Enrichment Analysis for IBS-Related Proteins

**Figure 2:**
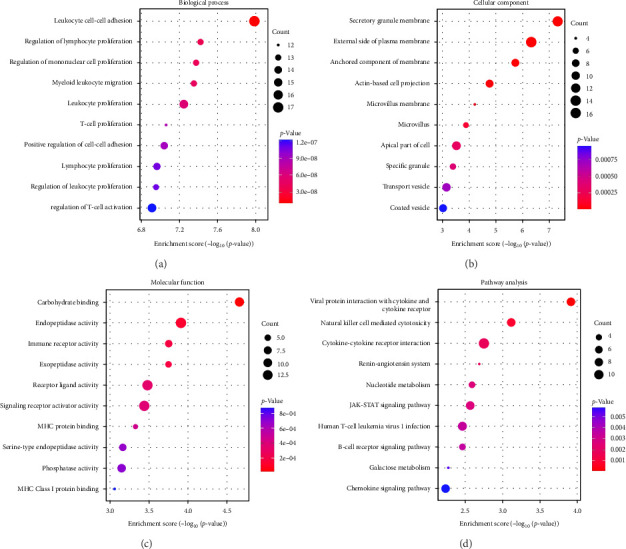
Gene Ontology (GO) and Kyoto Encyclopedia of Genes and Genomes (KEGG) enrichment analysis results for 169 proteins. (A) Bubble plots for the top 10 enrichment terms in Biological Process (BP). (B) Bubble plots for the top 10 enrichment terms in cellular component (CC). (C) Bubble plots for the top 10 enrichment terms in molecular function (MF), respectively. (D) Bubble plot of the top 10 KEGG enrichment analysis results.

GO-enrichment analysis shows that the pathogenesis of IBS is linked to key BPs such as leukocyte adhesion, lymphocyte and mononuclear cell proliferation, myeloid leukocyte migration, and T-cell activation, all of which are integral to immune response and regulation (Figure 2A). The cellular components (CCs) implicated in IBS include secretory granule membranes, plasma membrane regions, membrane-anchored components, actin-based cell projections, and microvillus membranes, reflecting the cellular structures that mediate immune functions (Figure 2B). MFs associated with IBS involve carbohydrate binding, various peptidase activities, immune receptor functions, receptor–ligand interactions, signaling receptor activation, and major histocompatibility complex (MHC) protein binding, indicating the diverse molecular mechanisms underlying immune recognition and signaling in IBS (Figure 2C).

The KEGG enrichment analysis highlights the potential involvement of various biological pathways in the pathogenesis of IBS (Figure 2D). These pathways encompass interactions between viral proteins and cytokines, natural killer cell cytotoxicity, cytokine signaling, the renin (REN)–angiotensin system, nucleotide metabolism, and the Janus Kinase-Signal Transducer and Activator of Transcription (JAK-STAT) signaling pathway, underscoring the complex interplay of immune and metabolic processes in IBS.

### 3.3. PPI Networks and Filtering Core Proteins

**Figure 3:**
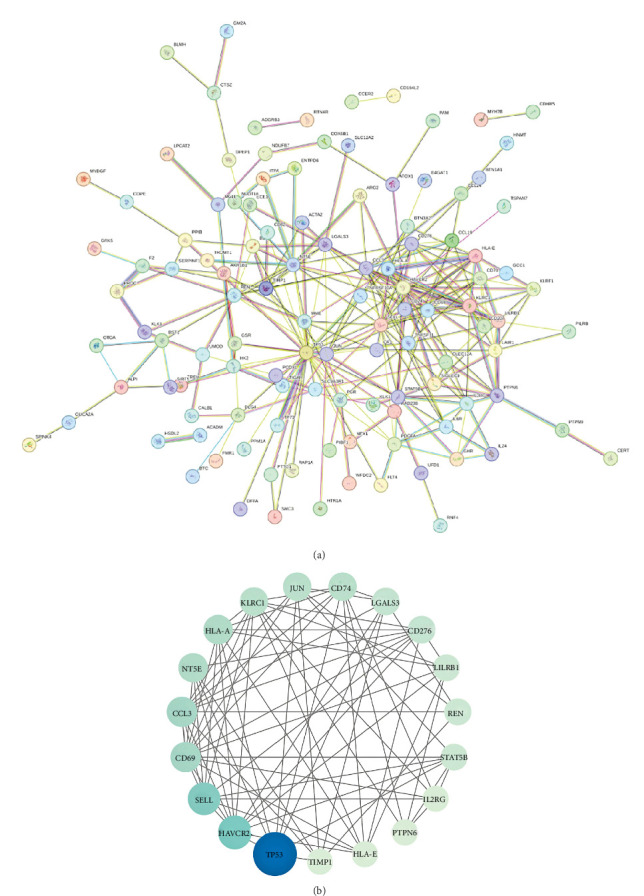
Protein–protein interaction (PPI) network for proteins. (A) After removing proteins with no interactions with other proteins, the PPI network retained 114 proteins. (B) A total of 19 proteins were selected based on a degree of at least 10, with the darkness and size of the node color being proportional to the degree.

**Figure 4:**
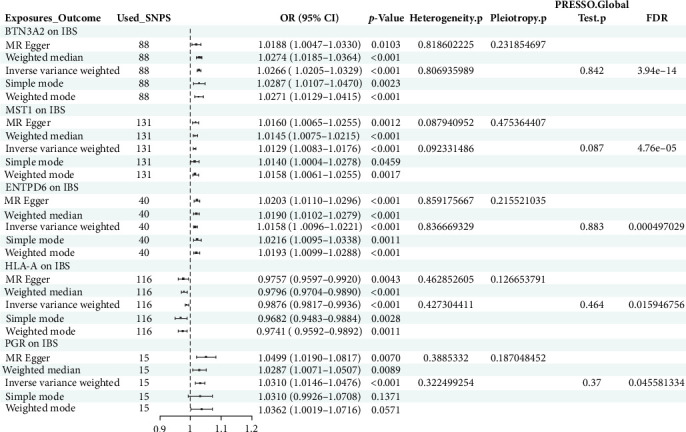
The forest plot illustrates the causal relationship between proteins and irritable bowel syndrome (IBS). 95% CI, 95% confidence interval; OR, odds ratio; PRESSO, pleiotropy residual sum and outlier; SNPs, single-nucleotide polymorphisms.

After analyzing the interactions among 169 proteins using the String database, we removed those proteins that did not interact with other proteins, ultimately retaining 114 proteins. Subsequently, we performed a topological analysis of these proteins using Cytoscape software (version 3.10.1). During the analysis, we screened out 19 core proteins based on the criterion of a degree greater than or equal to 10 (Figure 3).  Figure 4 shows the causal relationships between these 19 core proteins and IBS.

### 3.4. Acquisition of Core Protein-Related Drugs

**Figure 5:**
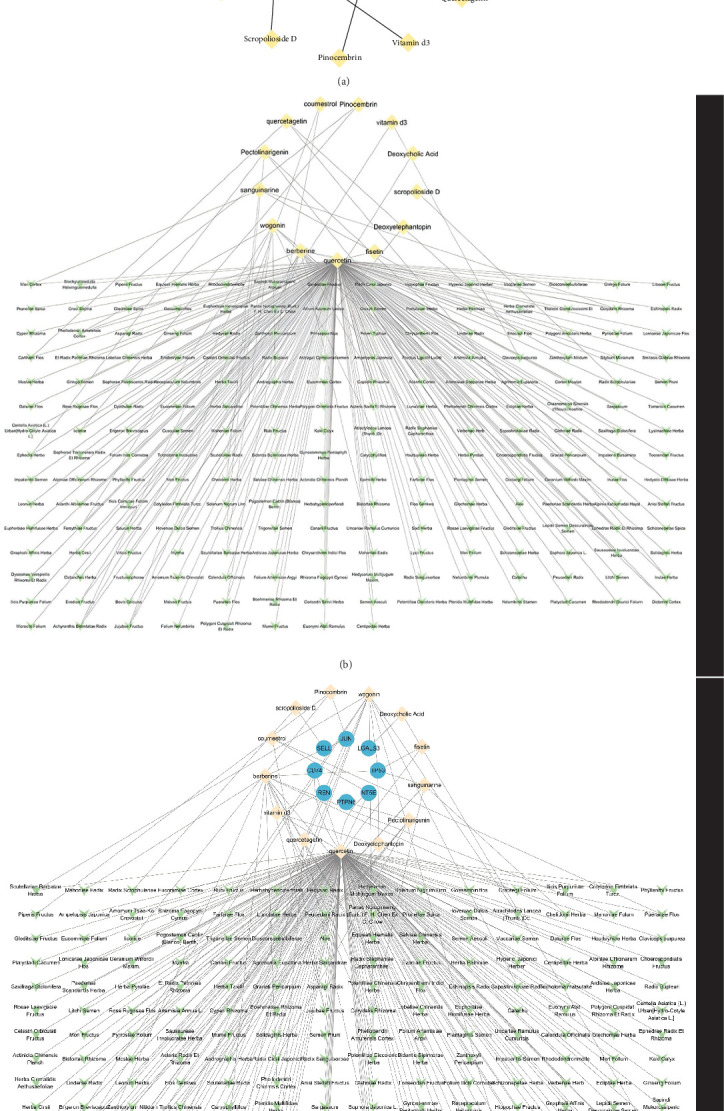
Network for core proteins, bioactive compounds, and traditional Chinese medicines. (A) Bioactive compound-core protein network. (B) Bioactive compound-traditional Chinese medicine network. (C) Core protein-bioactive compound-traditional Chinese medicine network. Circular blue nodes represent core proteins, diamond yellow nodes represent bioactive compounds, and V-shaped green nodes represent traditional Chinese medicines.

**Figure 6:**
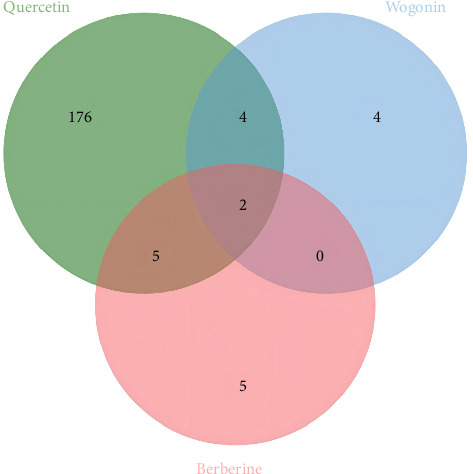
Venn diagram for core compounds and their related traditional Chinese medicines.

Leveraging the TCMSP database and the Herb database, we searched for active compounds that interact with these 19 core proteins. After filtering based on the criteria of OB ≥30%, DL ≥0.18, and HL ≥4 h, we retained 13 active compounds corresponding to 204 traditional Chinese medicines related to eight core proteins. The eight core proteins include Tumor Protein p53 (TP53), selectin L (SELL), REN, protein tyrosine phosphatase, nonreceptor type 6 (PTPN6), 5′ (3′)-deoxyribonuclease (NT5E), galectin-3 (LGALS3), Jun Proto-Oncogene (JUN), and MHC, Class II Invariant Chain (CD74).  Figure 5C presents the core protein-bioactive compound-traditional Chinese medicine network. Subsequently, we performed topological analysis on both the bioactive compound-core protein network (Figure 5A) and bioactive compound-traditional Chinese medicine network (Figure 5B). By filtering for active compounds with a degree ≥1 in the bioactive compound-core protein network and a degree ≥10 in the bioactive compound-traditional Chinese medicine network, we identified quercetin, berberine, and wogonin as potentially effective core compounds (Table 1). Using Venn diagrams, we found two traditional Chinese medicines containing all three core active compounds: Phellodendri Amurensis Cortex (PAC) and Achyranthis Bidentatae Radix (ABR) (Figure 6).

### 3.5. Molecular Docking Results

We have chosen core compounds and their corresponding core proteins for molecular docking, and the results show that the binding energy of each protein with each compound is less than −5 kcal/mol, indicating that there is good binding activity between each protein and compound (Table 2).

### 3.6. MD Simulation Results

**Figure 7:**
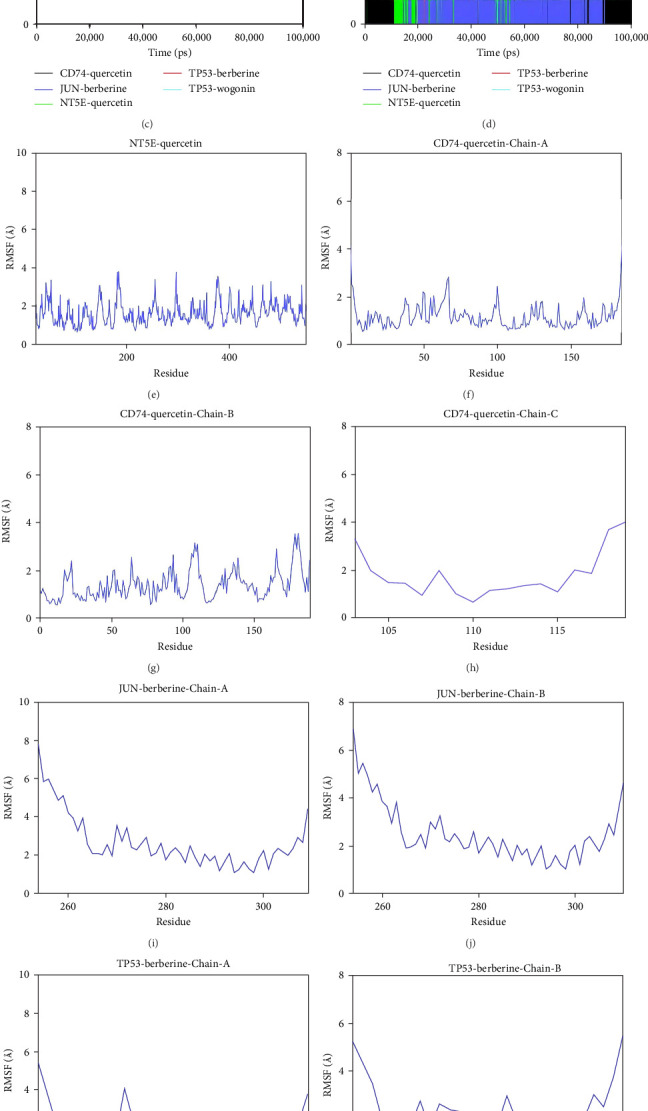
Molecular dynamics simulation results. (A) RMSD analysis. (B) Rg analysis. (C) SASA analysis. (D) Number of hydrogen bonds in five systems. (E–N) RMSF analysis. Rg, radius of gyration; RMSD, root mean square deviation; RMSF, root mean square fluctuation; SASA, solvent-accessible surface area.

The root mean square deviation (RMSD) is a good indicator for measuring the conformational stability of proteins and ligands, as well as the degree of deviation of atomic positions from their initial positions. The smaller the deviation, the better the conformational stability. Therefore, the equilibrium of the simulation system was assessed using RMSD (Figure 7A). The CD74-quercetin complex system reached equilibrium after 10 ns, with fluctuations around 2 Å. The TP53-wogonin complex system reached equilibrium after 20 ns, with fluctuations around 5.1 Å. The JUN-berberine complex system reached equilibrium after 20 ns, with fluctuations around 2.3 Å. The NT5E-quercetin complex system reached equilibrium after 30 ns, with fluctuations around 3.2 Å. The TP53-berberine complex system reached equilibrium after 20 ns, with fluctuations around 4.2 Å. Among them, the CD74-quercetin complex has the lowest RMSD value, indicating that the CD74-quercetin complex exhibits high stability.

Further analysis revealed that the radius of gyration (Rg) and the solvent-accessible surface area (SASA) of the CD74-quercetin, TP53-wogonin, JUN-berberine, NT5E-quercetin, and TP53-berberine complexes fluctuated stably during the motion, indicating that the target protein-small molecule complexes remained stable and compact throughout the simulation process (Figure 7B,C).

Hydrogen bonds play a significant role in the binding of ligands to proteins. The number of hydrogen bonds between the small molecules and target proteins during the dynamics is shown in Figure 7D. For the CD74-quercetin complex, the number of hydrogen bonds ranged from 0 to 5, with the complex typically having about three hydrogen bonds in most cases. For the TP53-wogonin complex, the number of hydrogen bonds ranged from 0 to 3, with the complex typically having about two hydrogen bonds in most cases. For the JUN-berberine complex, the number of hydrogen bonds ranged from 0 to 2, with the complex typically having about one hydrogen bond in most cases. For the NT5E-quercetin complex, the number of hydrogen bonds ranged from 0 to 6, with the complex typically having about four hydrogen bonds in most cases. For the TP53-berberine complex, the number of hydrogen bonds ranged from 0 to 3, with the complex typically having about two hydrogen bonds in most cases. This suggests that the CD74-quercetin, TP53-wogonin, JUN-berberine, UN-berberine, and NT5E-quercetin complexes have good hydrogen bond interactions.

The root mean square fluctuation (RMSF) can represent the flexibility of amino acid residues in proteins. As shown in Figure 7E–N, the RMSF values for the CD74-quercetin complex are mostly between 0.5 and 2.0 Å, for the TP53-wogonin complex they are mostly between 1.6 and 2.8 Å, for the JUN–berberine complex they are mostly between 1.2 and 2.5 Å, for the NT5E-quercetin complex they are mostly between 1.1 and 2.3 Å, and for the TP53-berberine complex they are mostly between 1.3 and 1.8 Å. Among them, the RMSF values of the CD74-quercetin complex are relatively low, indicating that it has lower flexibility and higher stability.

In summary, this group of small molecules interacts well with the target proteins. Notably, the CD74-quercetin complex exhibits more stable binding, and the complex has good hydrogen bonding interactions.

## 4. Discussion

IBS is a chronic functional gastrointestinal disorder characterized by abdominal discomfort or pain related to defecation and a change in bowel habits, without evidence of organic lesions [35]. The treatment of IBS usually includes dietary adjustments, behavioral therapy, pharmacological treatment, psychotherapy, traditional medicine, allergen desensitization, antibiotic treatment, alternative medicine, etc. [36]. Future research areas are expected to cover the search for more efficient pharmacotherapies, in-depth exploration of the potential impact of the gut microbiota, and the study of targeted strategies for cytokine pathways. Given the complexity of IBS and the diversity of its symptoms, a multifaceted, interdisciplinary research approach is necessary to deepen our understanding of its pathophysiological mechanisms and to develop more precise and effective treatment methods.

Currently, proteomics has been widely applied in the study of inflammatory diseases, immune diseases, metabolic diseases, and cancer, aiding in the in-depth understanding of disease mechanisms, the discovery of biomarkers, and the development of new therapies [37–40]. In this study, we employed MR methods combined with proteomics data of 2941 proteins to conduct an in-depth causality assessment of IBS. Through this analysis, we identified 169 potential proteins related to IBS. Proteomics plays an irreplaceable role in the clinical field of IBS. It not only reveals disease biomarkers, providing a basis for screening, diagnosis, and prognosis, but also, against the backdrop of relatively limited current treatment options, an in-depth understanding of the intrinsic links between protein changes and IBS is of profound significance for discovering new therapeutic targets and developing more precise treatment plans. The gut, as the primary site of IBS pathological changes, is also a key component of peripheral protein metabolism, and its protein expression patterns may be altered by inflammatory responses. Moreover, systemic inflammation associated with IBS may indirectly affect protein metabolism in multiple tissues, including the gut and the central nervous system, through a series of complex inflammatory pathways [41]. Therefore, exploring the potential interactions between IBS and proteins in depth is crucial for innovation in future treatment strategies.

We performed GO enrichment on these 169 proteins and found that the characteristic of IBS lies in its complex immune regulatory network, involving the adhesion, proliferation, and migration of immune cells, as well as the fine-tuning of immune responses. These processes occur in specific cellular structures, such as the secretory granule membrane and microvillus membrane, which are crucial for immune function. Furthermore, the diversity of MFs in IBS, including key molecular activities in immune recognition and signal transduction, further emphasizes the complexity of immune regulation and cell-to-cell communication involved in the disease.

KEGG enrichment analysis suggests that the pathophysiology of IBS involves the activation of the immune system, intercellular signal transmission, and the regulation of metabolic pathways. The interaction between viral proteins and cytokines and receptors, cytokine–cytokine receptor interactions, natural killer cell-mediated cytotoxicity, B-cell signaling pathways, and JAK-STAT signaling pathways are all potential etiological factors of IBS.

Severe acute respiratory syndrome coronavirus 2 (SARS-CoV-2) shedding through the gastrointestinal tract leads to epithelial barrier damage, immune activation, changes in microbiota composition, dysregulation of the serotonin pathway, and intestinal neuropathy, which may be significant factors contributing to the high incidence of IBS in patients during acute infection and postrecovery [42]. Additionally, Metagenomic analysis reveals that the gut virome of IBS patients is significantly different from that of healthy individuals, with both sample diversity and the abundance of 127 viral operational taxonomic units (vOTUs) showing significant differences [43]. These results suggest the important role of viral infections in the pathogenesis of IBS. It is noteworthy that since our results are based on MR analysis, this suggests a potential causal relationship between viral infections and the occurrence of IBS. However, further MR analyses targeting the causal relationship between viruses and IBS, as well as experimental validation, are needed to prove the correlation.

Based on the results of KEGG enrichment analysis, there may be a causal link between the B-cell signaling pathway and IBS. Specifically, the study found that in patients with diarrhea-predominant IBS, there is an enhancement of the humoral immune response in the jejunum, which is closely related to clinical manifestations [44]. Additionally, specific immunoglobulin E (IgE)-dependent mast cell activation was observed in the colonic mucosa of IBS patients, which may play a key role in food-induced abdominal pain [44]. Integrating these research findings, we can infer that the enhancement of humoral immune responses and specific IgE-dependent mast cell activation in diarrhea-predominant IBS (IBS-D) patients may be closely associated with the emergence of IBS symptoms.

The activity of the REN–angiotensin system may be involved in the pathogenesis of IBS. Under the influence of long-term chronic stress, the inflammatory response of the hypothalamic–pituitary–adrenal (HPA) axis may induce IBS [45]. Animal experiments have shown that inhibiting the expression of corticotropin-releasing hormone receptor 1 (CRF1), thereby suppressing the activity of the HPA axis, may be an effective method to alleviate diarrhea symptoms in IBS [46]. Our results further suggest that the REN–angiotensin system may have a direct causal relationship with IBS.

Our research results suggest that the JAK-STAT signaling pathway may be closely related to the occurrence of IBS. In patients with IBS-D and stress model mice, there is a significant increase in xanthine levels in the serum metabolome [47]. Concurrently, type I interferon (IFN-I) shows a marked decrease in the colonic tissue of these mice under the influence of stress and xanthine, suggesting that the dysfunction of the IFN-I signaling pathway may be associated with the development of IBS-D [47]. The transmission of interferon signals relies on the JAK-STAT signaling pathway, a process that begins at the receptor level and involves members of the Janus kinase family (including JAK and TYK) and STAT proteins, ultimately leading to the transcriptional activation of interferon-stimulated genes (ISGs) [48]. These conclusions further highlight the importance of exploring the connection between the JAK-STAT signaling pathway and IBS.

Through PPI network and topological analysis, we identified 19 core proteins out of the 169 proteins, and through reverse network pharmacology and rigorous filtering, we retained eight core proteins and 13 active compounds that have a relationship with them, which are present in 204 traditional Chinese medicines. By further applying topological analysis to the active compounds, we determined that quercetin, berberine, and wogonin may be key compounds for the treatment of IBS, while PAC and ABR may be potentially effective traditional Chinese medicines for the treatment of IBS. Additionally, we conducted molecular docking and MD simulations, and the results indicated that each protein has good binding stability with its corresponding compound.

Preclinical studies have found that quercetin shows potential therapeutic effects on postinflammatory IBS (PI-IBS) by reducing 5-hydroxytryptamine (5-HT) levels and regulating the differentiation of enteroendocrine cells, increasing pain thresholds, and reducing visceral motor responses [49]. Clinical trials have indicated that supplementation with quercetin can significantly decrease various inflammatory markers in healthy subjects (with mild dysbiosis of the gut microbiota), such as interleukin-6/interleukin-1β (IL-6/IL-1β) and tumor necrosis factor-α (TNF-α), and also reduce oxidative markers [50]. However, the effectiveness of quercetin in treating IBS still requires further research and exploration. In clinical trials, compared to placebo, berberine hydrochloride significantly reduced the frequency of diarrhea, abdominal pain, and the urgent need for bowel movements, and improved IBS symptoms, depression, anxiety, and quality of life scores [51]. Observational studies have shown that the combined use of berberine and curcumin may have potential synergistic pharmacological effects, significantly improving IBS patients' symptoms such as abdominal pain, bloating, and intestinal transit, and enhancing their quality of life, while reducing the need for traditional IBS treatment drugs [52]. Animal experiments have indicated that berberine exhibits multifaceted effects in the treatment of IBS, including regulating the gut microbiota, improving gut–brain axis function, and potential mood-regulating effects [53]. These effects may work together to alleviate IBS symptoms. However, the potential mechanisms of berberine in treating IBS have not been fully elucidated.

Our research found that berberine may treat IBS by targeting TP53 and JUN, two proteins that have a potential causal relationship with the pathogenesis of IBS, but further experimental validation is still needed. Systematic reviews and network pharmacology studies suggest that wogonin, as one of the key chemical metabolites in traditional Chinese medicine formulations, may have potential therapeutic effects on IBS-D and its associated anxiety and depressive symptoms [54]. From a causal perspective, combined with reverse network pharmacology, we also found that wogonin may have potential for IBS treatment, but this conclusion still requires further experimental verification. The results suggest that quercetin, berberine, and wogonin all have the potential to treat IBS and provide a scientific basis for our intersection of traditional Chinese medicines associated with these three core active compounds.

PAC, also known as Guan Huang Bai, originates from the bark of *Phellodendron amurense* Rupr. Beyond its traditional effects of clearing heat and drying dampness, detoxifying, and reducing inflammation, modern studies have identified additional biological activities, such as antioxidant, hypoglycemic, antitumor, immunomodulatory, and neuroprotective properties [55]. ABR, commonly known as Niuxi, is the dried root of the plant *Achyranthes bidentata* Blume. It has been widely used in traditional medicine for the treatment of amenorrhea, dysmenorrhea, difficult labor, dizziness, and trauma, among other conditions [56]. Our research indicates that PAC and ABR may be potentially effective traditional Chinese medicines for the treatment of IBS, and the common active compounds of these two drugs, quercetin, berberine, and wogonin, may target proteins TP53, NT5E, JUN, and CD74, which have a positive causal relationship with IBS. However, this conclusion still requires further validation.

Despite employing various statistical methods to enhance the robustness of our research findings, such as conducting pleiotropy tests and heterogeneity tests to confirm the reliability of the results, our study still faces some notable limitations. First, our research only covered pQTL data for 2941 proteins, which may limit our ability to identify a broader range of protein expression variations. Second, the IBS samples we selected were predominantly from European populations, raising questions about the generalizability of our findings to other races or ethnic groups. In the network pharmacology section, we chose proteins with a degree value of 10 or higher based on topological analysis and applied stringent selection criteria for the active compounds interacting with these proteins, which may have led to the omission of some compounds that could be effective in treating IBS. Lastly, and most critically, our conclusions are based on predictions of causal relationships and database analyses, which require further experimental validation for support.

## 5. Conclusion

Our research integrates proteomics data and explores the possible etiology of IBS from a causal perspective. Additionally, we have newly discovered the potential of PAC and ABR, two traditional Chinese medicines, in treating IBS, as well as potential therapeutic targets. However, these conclusions require further validation.

## Figures and Tables

**Table 1:** Results of topological analysis of active compounds.

Proteins	Compounds	Binding energy (kcal/mol)
TP53	Wogonin	−5.9
TP53	Berberine	−6.6
NT5E	Quercetin	−7.6
JUN	Berberine	−8.5
CD74	Quercetin	−7.3

**Table 2:** Molecular docking results of three core active compounds with corresponding target protein molecules.

Compounds	Degree (compounds-herbs)	Degree (compounds-proteins)
Quercetin	187	2
Berberine	12	2
Wogonin	10	1
Sanguinarine	4	1
Pectolinarigenin	4	1
Quercetagetin	3	1
Pinocembrin	2	1
Coumestrol	2	1
Vitamin D3	2	1
Deoxyelephantopin	1	1
Fisetin	1	1
Deoxycholic acid	1	1
Scropolioside D	1	1

## Data Availability

The data that support the findings of this study are available from the corresponding author upon reasonable request.
